# Variability in BIA-Derived Muscle Mass Estimates: Device Choice Impacts Diagnostic Classification

**DOI:** 10.3390/nu18050767

**Published:** 2026-02-26

**Authors:** Leonie Cordelia Burgard, Siri Goldschmidt, Verena Alexia Ohse, Hans Joachim Herrmann, Dejan Reljic, Markus Friedrich Neurath, Yurdagül Zopf

**Affiliations:** 1 Hector-Center for Nutrition, Exercise and Sports, Department of Medicine 1, University Hospital Erlangen, Friedrich-Alexander University Erlangen-Nürnberg, 91054 Erlangen, Germany; 2 Department of Medicine 1, University Hospital Erlangen, Friedrich-Alexander University Erlangen-Nürnberg, 91054 Erlangen, Germany; 3 Deutsches Zentrum Immuntherapie (DZI), University Hospital Erlangen, Friedrich-Alexander University Erlangen-Nürnberg, 91054 Erlangen, Germany

**Keywords:** bioelectrical impedance analysis, muscle mass assessment, sarcopenic obesity, malnutrition, GLIM criteria

## Abstract

*Background/Objectives*: Although discrepancies between bioelectrical impedance analysis (BIA) devices are well documented, their clinical relevance in vulnerable populations remains unclear. This study aims to assess the impact of device choice on muscle mass classification criteria in patients with cancer or obesity and to identify modifiers of device variability. *Methods*: BIA data from 224 adults (85 with cancer, 139 with obesity) measured with two segmental multi-frequency devices (seca mBCA 515 and InBody 970) were analyzed. Device differences were assessed using the Wilcoxon signed-rank test and agreement analyses. Differences in classification of body composition cut-offs cited in the GLIM criteria for malnutrition and the ESPEN and EASO criteria for sarcopenic obesity were evaluated using McNemar’s test. The impact of disease type, sex, and age on device differences was examined through multivariable models. *Results*: Significant device differences were found for all parameters (all *p* ≤ 0.005). Discrepancies were largest for skeletal muscle mass (kg and %), with effect sizes r > 0.8 and poor agreement (Lin’s CCC < 0.90). A significant impact of device choice on muscle mass classification was observed for both cancer and obesity patients (*p* < 0.001), with seca classifying more patients as having low fat-free mass (50% vs. 20%) and as having a body composition consistent with sarcopenic obesity (90% vs. 50%) than InBody. Discrepancies were more pronounced in cancer patients and females. *Conclusions*: Muscle mass assessment by BIA is highly dependent on device choice, potentially leading to clinically relevant discrepancies in classification when rigid cut-offs are applied. An individualized interpretation of BIA data and further validation of prediction equations in disease-specific subpopulations is warranted.

## 1. Introduction

Skeletal muscle is widely acknowledged as a significant contributor to health and disease, with its role extending far beyond the traditionally attributed functions related to movement, posture, and respiration. Today, muscles are recognized as key mediators of metabolic processes, with muscle-produced secretory proteins, referred to as myokines, playing a crucial role in cross-organ communication [1]. This makes skeletal muscle a key modifier in fundamental aspects of numerous diseases—from onset through progression to mortality. This is particularly evident in chronic inflammatory diseases, which are frequently associated with skeletal muscle wasting and dysfunction. Pro-inflammatory cytokines, such as IL-6 and TNF-α, can contribute to catabolic signaling pathways and impaired protein synthesis under conditions of sustained inflammation, thereby affecting muscle integrity and function [2]. Skeletal muscle exhibits immunomodulatory properties itself [1,3], and engages in bidirectional crosstalk with the immune system, which may facilitate inflammatory responses in chronic disease [4].

Accurate assessment of muscle mass is therefore crucial for guiding therapeutic decisions and monitoring disease progression in both clinical and research settings. Computed tomography (CT) and magnetic resonance imaging (MRI) are widely regarded as the gold standards for non-invasive muscle mass assessment, as they allow for direct quantitative and qualitative measurement of skeletal muscle mass (SMM). In clinical and research practice, CT is often preferred over MRI due to greater standardization and broader availability of reference data. However, the use of both CT and MRI in routine practice is limited by high acquisition costs, time-intensive procedures, the need for highly skilled personnel, and—in the case of CT—radiation exposure [5,6]. Dual-energy X-ray absorptiometry (DXA) represents a widely used and more accessible alternative, yet remains constrained by equipment availability, staffing requirements, and radiation—limiting its feasibility for frequent assessments.

Against this background, bioelectrical impedance analysis (BIA) is often selected as the method of choice: it requires minimal technical expertise, is inexpensive, radiation-free, quick to perform, and available in portable formats [6]. Unlike imaging-based methods, BIA provides an indirect estimate of body composition parameters by measuring the body’s impedance to a low-amplitude electrical current. These impedance values are subsequently transformed into estimates of total body water (TBW) and fat-free mass (FFM). SMM is then derived from FFM using prediction equations that incorporate assumptions regarding tissue conductivity, hydration, and population characteristics [7].

Because these prediction equations are device specific, BIA outputs may differ not only between manufacturers but also across models of the same brand [8]. Consequently, measurement accuracy depends on how closely a patient resembles the specific population from which the prediction equations were derived [8]. As most equations originate from generally healthy populations, their validity may be reduced in clinical settings [8]. This may be particularly the case in diseases frequently associated with alterations in body fluid distribution, such as cancer and obesity [9,10], given that BIA relies on assumptions regarding FFM hydration and the ratio of extracellular to intracellular water [9]. Deviations from standard physiological models and hence device-related variability may therefore be more pronounced in these patient groups.

Although BIA is routinely used in clinical decision-making and numerous studies have identified device-related discrepancies, the clinical implications of such variability remain insufficiently understood. This is of concern because current diagnostic frameworks—such as the Global Leadership Initiative on Malnutrition (GLIM) criteria for malnutrition and the European Society for Clinical Nutrition and Metabolism (ESPEN) and the European Association for the Study of Obesity (EASO) cut-offs for sarcopenic obesity—list BIA as a measurement method for applying diagnostic thresholds. If different devices provide systematically divergent estimates, diagnostic outcomes may vary accordingly, potentially affecting the consistency and reliability of guideline-recommended cut-offs.

The aim of this study is therefore (i) to compare body composition outputs between two widely used multifrequency segmental BIA devices in patients with cancer and obesity, (ii) to evaluate the clinical relevance of potential device discrepancies by assessing whether they lead to divergent diagnostic classifications of muscle mass criteria, and (iii) to assess whether device variability is influenced by disease type (cancer/obesity), sex, or age group.

## 2. Subjects and Methods

This study is a cross-sectional secondary analysis of two randomized controlled trials conducted at the Hector-Center for Nutrition, Exercise and Sports at the University Hospital Erlangen, Germany. The sample includes adult patients of European descent (20–80 years) with obesity (BMI ≥ 30 kg/m^2^) or cancer who provided consent for the use of their data in secondary analyses. Patients who met both criteria (i.e., with concomitant obesity and cancer) were excluded to ensure a clear distinction between groups. Apart from this, no additional exclusion criteria beyond those of the original trials were applied. Data were collected between 2022 and 2025. Only one measurement time point per participant was included. If multiple assessments were available, the chronologically first measurement was selected. Further details of the primary studies can be obtained from the corresponding clinical trial registry entries (see NCT06885255 and NCT04067167).

The studies received approval from the Ethics Committee of the Friedrich-Alexander-Universität Erlangen-Nürnberg (FAU) (22-60-B and 81-19-B). To ensure compliance with quality standards in health research reporting, the STROBE guidelines were followed during the writing of this manuscript [11].

Given that this study represents a secondary analysis of existing trials, no a priori sample size calculation was performed. The sample size of *n* = 224 thus reflects all eligible participants with duplicate BIA measurements collected within the study period, ensuring maximal data inclusion. According to recently published domain-specific effect size guidelines, the present sample size provides sufficient statistical power (>90%) to detect medium between-group effects at a two-sided α level of 0.05 [12].

Anthropometric measurements included waist circumference, body height, and BIA. All measurements were performed at the study center by trained personnel following manufacturers’ instructions. Waist circumference was measured at the midpoint between the lowest rib and the iliac crest, and hip circumference at the level of the greater trochanter. During measurements, participants stood with their feet hip-width apart and were instructed to exhale. Body height was measured using a stadiometer (seca 274, seca GmbH & Co., KG, Hamburg, Germany). All values were recorded to the nearest 0.5 cm.

BIA measurements were performed using two commercial segmental multi-frequency devices: the mBCA 515 (seca GmbH & Co. KG, Germany) and the InBody 970 (InBody Co., Ltd., Seoul, Republic of Korea), hereafter referred to as seca and InBody, respectively. The seca mBCA 515 has been validated against whole-body MRI, four-compartment models, and dilution methods across multi-ethnic samples [13,14,15]. The InBody 970 has primarily been validated against DXA [16,17]. Both systems conduct standing measurements using eight electrodes but differ in their frequency ranges and applied currents. The InBody device uses a frequency spectrum ranging from 1 kHz to 3000 kHz, with current values of 70 µA (±10 µA) at 1 kHz and 300 µA (±30 µA) from 5 kHz onwards. The seca device operates on a frequency spectrum from 1 kHz to 1000 kHz with a current of 100 µA (+20%, −50%) [18,19]. Both devices mandate manual input of sex, height, and date of birth, whereas only the seca interface additionally requires the entry of waist circumference and ethnicity. The predictive equations embedded in the seca system are partly disclosed by the manufacturer [19], whereas those used by InBody are proprietary and not published by the manufacturer [20]. Further technical specifications are available in the manufacturers’ handbooks [18,19].

The two BIA measurements were performed immediately after one another in random order, without a rest period between measurements. Prior to measurements, participants rested seated for at least five minutes to stabilize body fluid distribution and minimize external influences on conductivity. Measurements were conducted barefoot and in indoor clothing, with pockets emptied prior to assessment. To account for clothing weight, a standardized deduction of 0.5 kg was specified for both devices. In accordance with best-practice protocols, patients fasted for at least eight hours before measurements [21] and were further instructed to refrain from any strenuous physical activity and only have the measurement taken if there were no symptoms of acute illness.

With regard to the variables analyzed, the study focuses on established clinical markers: FFM, SMM, TBW, and body fat (BF). Additionally, resistance, reactance, and PhA are included to help detect potential hardware-related differences.

To evaluate whether discrepancies between devices lead to different muscle mass classification of body composition criteria, BIA measurements were compared against established clinical cut-offs. For patients with cancer, the fat-free mass index (FFMI = FFM/height^2^) was evaluated using FFMI thresholds cited in the GLIM consensus report (FFMI < 17 kg/m^2^ for men and <15 kg/m^2^ for women) [22,23]. For patients with obesity, joint reference values for sarcopenic obesity from ESPEN and EASO were used [24]. According to this guidance, sarcopenic obesity is defined as the coexistence of obesity with both altered skeletal muscle function and altered body composition. When assessed by BIA, altered body composition is defined as increased BF percentage together with reduced SMM adjusted for body weight. The specific cut-offs used for classification in this study are listed in Table 1. Based on these criteria, patients were classified as either below or above the respective cut-off. Only BIA-assessable body composition criteria were evaluated; additional phenotypic, etiologic, or functional components required for formal diagnostic classification were not assessed. Neither GLIM nor ESPEN/EASO prescribe a single measurement modality or universal thresholds. The cut-offs applied in this study are commonly used BIA-derived reference values cited in the respective consensus documents.

Statistical analyses were performed using SAS version 9.4 [27]. Descriptive statistics are presented as means ± standard deviation for continuous variables and as absolute (*n*) and relative (%) frequencies for categorical variables. Data distribution was examined using Q–Q plots and the Shapiro–Wilk and Kolmogorov–Smirnov tests. As most variables were not normally distributed, between-device differences were analyzed using the Wilcoxon signed-rank test. Effect sizes were calculated according to Rosenthal’s r (r = Z/√N) [28], where Z is the Wilcoxon test statistic and N the number of non-zero differences. Agreement between parameters obtained from the two BIA devices was assessed using Lin’s concordance correlation coefficient (CCC) with 95% confidence intervals and visualized by Bland–Altman plots [29]. To examine whether discrepancies between devices affected diagnostic classification of body composition criteria, McNemar’s test was applied. To account for agreement beyond chance, Cohen’s kappa (κ) was calculated and interpreted according to Landis and Koch [30]. Device variability by disease type, sex, and age group was assessed using multivariable linear models with device differences (InBody-seca) as dependent variables. All covariates were included in a single model to estimate their independent associations with device differences while adjusting for potential confounding. A significance level of *p* < 0.05 was set for all analyses.

## 3. Results

### 3.1. Study Sample

As displayed in Table 2, the total sample comprises 224 participants, either diagnosed with cancer (*n* = 85) or obesity (*n* = 139). The majority of participants were female and middle-aged. Compared with the obesity group, the cancer group included relatively older participants and a higher proportion of females. Among participants with cancer, breast cancer (41.2%), gastrointestinal cancers (21.2%), and other gynecological cancers (11.8%) were the most common entities, as detailed in Supplementary Table S1.

### 3.2. Between-Device Comparison and Agreement

**Figure 1:**
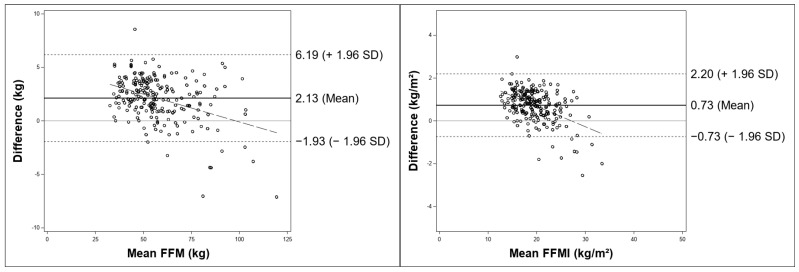

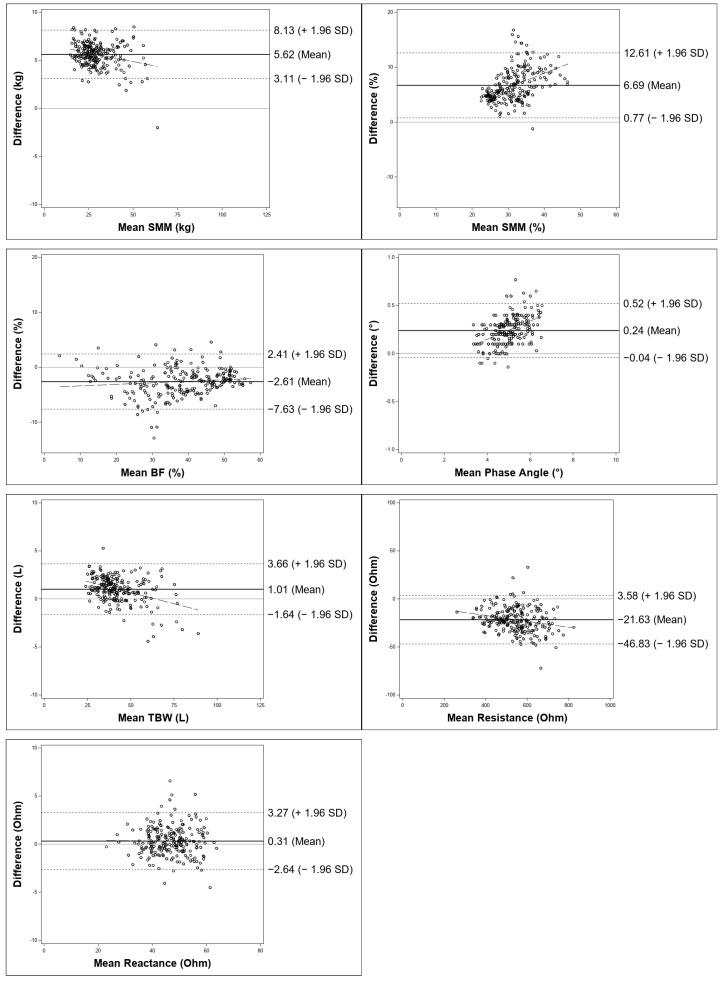
Bland–Altman plots illustrating between-device agreement for BIA parameters measured by the InBody 970 and seca mBCA 515 devices in the total sample (*n* = 224). Solid line = mean bias; dotted lines = 95% limits of agreement (mean ± 1.96 SD); dashed lines = regression line. Note: For reactance, the regression line is overlain by the solid line representing the mean bias. FFM = fat-free mass; FFMI = fat-free mass index; SMM = skeletal muscle mass; BF = body fat; TBW = total body water.

Descriptive, inferential, and agreement analyses were performed to evaluate the differences between seca and InBody measurements. Wilcoxon signed-rank tests revealed significant differences in all BIA parameters analyzed, with the largest effects observed for SMM (kg and %). As shown in Table 3, InBody yielded higher mean values than seca for all parameters analyzed except for BF (%) and resistance (Ω). According to Lin’s concordance correlation coefficients displayed in Table 4, the agreement between InBody and seca ranged from poor for SMM (kg and %) to almost perfect for TBW (L). As illustrated in the Bland–Altman plots in Figure 1, the between-device differences were most pronounced for SMM (kg and %), with mean biases of +5.62 kg (95% LoA 3.11–8.13) and +6.67% (95% LoA 0.76–12.58), respectively. For SMM %, a proportional bias was observed, with larger differences at higher mean values. In contrast, the biases for FFM (kg), FFMI (kg/m^2^), BF (%), and particularly PhA (°), TBW (L), and resistance (Ω) were considerably smaller, indicating a closer agreement between the two devices. Reactance showed a negligible mean bias. A detailed summary of the authors’ interpretation of the Bland–Altman plots is provided in the supplement (see Supplementary Table S2).

### 3.3. Comparison of Diagnostic Classification of Body Composition Criteria

In the cancer subsample, the classification of low muscle mass according to the FFMI thresholds cited in the GLIM consensus report differed significantly depending on the measurement source (InBody vs. seca; χ^2^ = 26.0, *df* = 1, *p* < 0.001). Based on seca measurements, more than twice as many participants were classified as having low muscle mass compared with InBody (50% vs. 20%, see Table 5). A comparable result was obtained for the obese subsample (χ^2^ = 51.1, *df* = 1, *p* < 0.001). Using seca data, nearly 90% of participants were classified as having altered body composition, whereas InBody data yielded such a classification in only about half of the sample (see Table 6). Overall percent agreement between devices was 69.4% for the cancer subsample, corresponding to fair agreement according to Landis and Koch (Cohen’s κ = 0.39). In the obesity subsample, percent agreement was 60.4% and Cohen’s κ indicated only slight agreement (κ = 0.08), reflecting limited diagnostic concordance between devices.

### 3.4. Subgroup Analyses of Between-Device Differences

Table 7 summarizes the between-device differences across the subgroups. Device-related deltas (InBody-seca) were significantly larger in the cancer subsample than in the obesity subsample for all parameters except for PhA, FFM (kg), resistance, and reactance. For PhA, the delta was significantly larger in the obesity group, whereas no significant differences were found for FFM (kg), resistance, and reactance. With respect to sex, significant differences were observed for all parameters except SMM (kg), with females generally showing larger deltas than males. Regarding age groups, significant differences were found for SMM (%) and PhA. Absolute BIA outputs stratified by disease type, sex, and age group are provided in Supplementary Tables S4–S6.

## 4. Discussion

The findings of this study demonstrate that even technically comparable BIA devices can yield systematically different results. The strongest deviations were observed for skeletal muscle mass, limiting comparability between systems and challenging the assumption of device interchangeability. Notably, device discrepancies were reflected in divergent classifications of muscle mass criteria, with potential consequences for clinical decision-making. Moreover, device-related variation appeared to be influenced by disease status and demographic factors, suggesting a multifactorial and not readily predictable nature of measurement bias.

SMM emerged as the most vulnerable parameter, consistent with the hierarchical structure of BIA modeling, in which SMM is estimated through multiple sequential steps from resistance and reactance raw data to TBW, FFM, and ultimately SMM, allowing upstream variability to be amplified in downstream estimates. The presence of significant between-device differences in resistance, reactance, and PhA suggests that the observed variability in BIA outputs cannot be attributed to predictive equations alone but also reflects hardware-related influences. In line with this interpretation, Stratton et al. reported significant method effects for resistance, reactance, and PhA across multiple BIA devices, including InBody 770 and seca mBCA 515/514 [32].

Consistent with the presence of systematic between-device differences, previous studies comparing BIA-derived BF estimates with DXA identified differing patterns of deviation for InBody and seca [33,34]. The observations of these studies—higher PhA and lower BF% with InBody compared to seca—mirror the present results. However, direct comparability is limited, as these studies, including the work by Stratton et al., examined the InBody 770 rather than the 970 model, which uses fewer measurement frequencies and previous-generation software [35] and were conducted in predominantly healthy populations.

Although research supports systematic discrepancies between BIA devices, evidence in clinically vulnerable populations, such as patients with cancer and/or obesity, remains scarce. Guedes et al. assessed measurement differences between two tetrapolar single-frequency BIA devices in 116 hospitalized adult cancer patients and found poor agreement across all parameters, particularly for reactance and PhA [36]. Further studies by Froon-Torenstra et al. and Thajer et al. compared BIA devices in pediatric populations with cancer and obesity, respectively [37,38]. However, beyond the limited comparability between pediatric and adult populations, these studies did not assess technically comparable BIA devices.

To the best of the authors’ knowledge, this is one of the first studies to examine the clinical relevance of between-device differences in BIA measurements with regard to diagnostic classification. Ward (2019) [39] previously argued that “*the focus on statistically significant but small differences between methods can obscure operational equivalence and that such differences may be of minor clinical significance*”. In contrast, the present findings indicate that between-device discrepancies indeed have potential clinical implications: Compared with InBody, seca resulted in 75% higher classification rates of altered body composition consistent with sarcopenic obesity and 250% higher rates of low muscle mass when applying the BIA-derived FFMI thresholds referenced in the GLIM consensus report. These discrepancies were reflected not only in significantly different classification frequencies but also in limited agreement beyond chance. This challenges the assumption that technically comparable BIA devices can be used interchangeably in clinical practice, particularly when body composition estimates are applied within rigid dichotomous classification frameworks. From a methodological perspective, this analysis is particularly important, as high inferential statistical significance may be expected in large samples and can partly reflect systematic algorithmic offsets between devices.

Our study further suggests that these discrepancies may be particularly relevant for patients with cancer. While agreement was somewhat higher in the cancer subsample than in the obesity subsample, the absolute proportion of patients classified as having low muscle mass differed substantially depending on the device used. Moreover, analyses demonstrated that the magnitude of device-related deltas was markedly greater in the cancer subsample compared with the obesity subsample. This was observed not only for relative but also for absolute parameters, despite the patients with cancer being considerably leaner. Relative SMM in cancer patients differed by 9.67% between devices, a magnitude that highlights the potential for device disagreement to influence clinical decision-making. In this context, device-dependent differences may influence the identification of low muscle mass, potentially affecting referral decisions, eligibility for nutritional interventions, and the interpretation of longitudinal changes in both clinical practice and research settings. This concern is further supported by several studies comparing BIA with CT or MRI, which reported systematic overestimation of muscle mass in oncology populations [40,41,42]. The particular vulnerability may be explained by the hydration shifts typically observed in cancer patients, characterized by an increase in extracellular water and a decrease in intracellular water [43], which may be processed differently within device-specific algorithms and thereby contribute to between-device discrepancies. While hydration alterations are also frequently observed in obesity, their pattern and magnitude appear less consistent than in cancer.

The application of fixed FFMI cut-offs without explicit reference to the measurement device assumes a degree of cross-device equivalence that is not supported by the present data. BIA device-related uncertainty thereby adds to the existing uncertainty surrounding the applied cut-off thresholds. The FFMI cut-offs (<15 kg/m^2^ for women and <17 kg/m^2^ for men) cited in the GLIM consensus report are derived from historical reference data obtained using tetrapolar single-frequency BIA in an apparently healthy Swiss population. While conceptually sound, these reference data were established under methodological conditions that differ substantially from contemporary clinical BIA practice—where multifrequency, segmental devices are commonly applied—and predate current epidemiological trends in obesity [44]. As a result, the transferability of established FFMI cut-offs to current practice may be inherently limited.

Looking beyond disease-specific vulnerabilities, our analyses indicate the bias between BIA devices to be multifactorial, leading to substantial inter-individual variability that is hardly predictable. Specifically, between-device differences also appear to be sex-specific, with larger discrepancies observed in females despite lower absolute mean values. Possible explanations include sex-differences in fat mass distribution and cycle-related hydration shifts.

Overall, the multifaceted nature of device-dependent discrepancies underscores the limitations of rigid dichotomization into “below” versus “above” cut-off categories and argues for a more individualized interpretation of body composition data in clinical practice, with greater emphasis on changes over time and an awareness that nutritional risk in cancer and obesity is inherently multifactorial and not fully captured by body composition parameters alone. Accordingly, clinicians should strive for a more nuanced assessment of treatment need, i.e., taking into account the additional contribution of inflammatory, oxidative stress, and adipokine-mediated pathways [45], and ensure the consistent use of the same device in follow-up measurements. Beyond their clinical implications, our findings highlight the need for action in both research and industry. Calls for joint efforts towards the harmonization of BIA measurements date back to 1996, yet little progress has been achieved even decades later [39], despite growing recognition of the clinical relevance of muscle status and the expanding use of BIA in clinical practice. To improve the validity and reliability of BIA-derived estimates in specific clinical populations, further validation and refinement of existing prediction models across defined patient groups appear warranted. Ideally, future research should evaluate whether more modular prediction frameworks—allowing the integration of multiple interacting patient characteristics—can enhance robustness in routine clinical use. With regard to guideline consortia, increased transparency concerning the derivation and device dependence of cut-off values may help raise awareness of their inherent vulnerability and promote more cautious interpretation in clinical practice. Furthermore, continuous reference ranges that incorporate patient demographics, rather than rigid cut-off thresholds, should be explored more thoroughly, as research suggests this approach improves the accuracy of body composition assessments [46]. Additionally, the concept of continuous reference ranges aligns with the need for individualized, change-focused interpretations of BIA data.

This study has two main limitations. First, the absence of a gold-standard reference method (such as CT or MRI) precludes a definitive evaluation of the accuracy of the comparison between the seca and InBody devices. Without such ground truth as reference, it cannot be determined which device more closely reflects true body composition. Against this background, the findings should be interpreted with particular emphasis on device non-interchangeability and the sensitivity of dichotomous cut-offs to device-specific measurement differences. Second, consecutive BIA measurements were performed without an intervening rest period. Changes in body position, subtle fluid redistribution, and residual effects of prior current application may have influenced impedance values, particularly in multi-frequency devices. As the measurement order was not recorded, the potential influence of sequence effects on the results cannot be excluded. Third, potential confounders such as treatment status, edema or ascites could not be accounted for. Fourth, unequal group sizes represent an additional limitation from a statistical perspective. Of note, both the GLIM criteria and the ESPEN–EASO consensus represent multi-component diagnostic frameworks that require the additional fulfilment of phenotypic, etiologic, and/or functional criteria for a formal diagnosis of malnutrition or sarcopenic obesity. The present analysis focused solely on body composition criteria assessable by BIA. Consequently, the findings relate to device-dependent classification of individual criteria rather than definitive clinical diagnoses.

## 5. Conclusions

In conclusion, this study demonstrates that muscle mass assessment using BIA is highly dependent on the choice of device. Between-device variability may translate into differences in muscle mass categorization that could influence clinical decision-making, particularly when rigid cut-offs are applied. Taken together, these findings argue for an individualized, change-focused interpretation of BIA data in clinical practice, acknowledging that nutritional risk in cancer and obesity is inherently multifactorial and not fully captured by body composition parameters alone. Furthermore, they highlight the importance of clinician awareness of device-related uncertainty, especially in vulnerable patient populations, such as those with cancer, where device-dependent classification differences could affect clinical decisions. Future research should focus on validating and refining prediction equations in distinct patient groups and on more extensively accounting for the multifactorial determinants of BIA measurement. Additionally, assessing between-device bias in longitudinal studies is mandated. Such advances are vital to enhance the clinical usability of BIA and to mitigate the risk of inappropriate clinical decision-making arising from device-related measurement bias.

## Figures and Tables

**Table 1:** BIA cut-off values for sarcopenic obesity according to the ESPEN and EASO consensus.

	Male	Female
**Increased BF % ^1^** [25]		
20–39 years	>26.0%	>39.0%
40–59 years	>29.0%	>41.0%
60–79 years	>31.0%	>43.0%
**Diminished SMM % ^2^** [26]	≤37.0%	≤27.6%

ESPEN = European Society for Clinical Nutrition and Metabolism; EASO = European Association for the Study of Obesity; BF = body fat; SMM = skeletal muscle mass. Thresholds from the Supplementary Materials of Donini et al., 2022 [24]; no perfectly matching reference group was available for SMM%; therefore, we used the closest available reference. ^1^ Referring to adults of white descent. ^2^ Referring to adults of mixed ethnic descent.

**Table 2:** Sample characteristics.

	Cancer (*n* = 85)	Obesity (*n* = 139)
Sex *n* (%)		
Male	24 (28.2)	54 (38.8)
Female	61 (71.8)	85 (61.2)
Age (years) mean ± SD	55.7 ± 12.2	51.3 ± 11.9
Age group in years *n* (%)		
20–39	9 (10.6)	23 (16.5)
40–59	41 (48.2)	81 (58.3)
60–80	35 (41.2)	35 (25.2)
Body height (cm) mean ± SD	169.6 ± 9.0	170.6 ± 10.4
Body weight (kg) mean ± SD	65.8 ± 10.5	113.2 ± 25.7
Waist circumference (cm) mean ± SD	81.8 ± 10.2	117.2 ± 16.0
BMI (kg/m^2^) mean ± SD	22.8 ± 3.0	38.7 ± 6.8
BMI categories *n* (%)		
Underweight (<18.5 kg/m^2^)	6 (7.1)	-
Normal weight (18.5–24.9 kg/m^2^)	57 (67.1)	-
Overweight (25.0–29.9 kg/m^2^)	22 (25.9)	-
Obesity class I (30–34.9 kg/m^2^)	-	48 (34.5)
Obesity class II (35–39.9 kg/m^2^)	-	40 (28.8)
Obesity class III (≥40.0 kg/m^2^)	-	51 (36.7)

BMI = body mass index.

**Table 3:** Descriptive statistics and Wilcoxon signed-rank test results for between-device differences in BIA parameters derived from the InBody 970 and seca mBCA 515 in the total sample (*n* = 224).

Mean ± SD	InBody 970	Seca mBCA 515	*p*-Value ^1^	Effect Size r ^2^
FFM (kg)	58.25 ± 15.39	56.12 ± 16.21	**<0.001**	0.74
FFMI (kg/m^2^)	19.86 ± 3.62	19.13 ± 3.98	**<0.001**	0.71
SMM (kg)	32.20 ± 9.07	26.58 ± 9.41	**<0.001**	0.87
SMM (%)	34.85 ± 5.92	28.18 ± 4.65	**<0.001**	0.87
BF (%)	36.62 ± 11.14	39.23 ± 10.83	**<0.001**	−0.75
PhA (°)	5.08 ± 0.75	4.84 ± 0.68	**<0.001**	0.79
TBW (L)	42.87 ± 11.40	41.86 ± 11.93	**<0.001**	0.63
Resistance (Ω)	534.09 ± 94.24	555.72 ± 97.16	**<0.001**	−0.84
Reactance (Ω)	46.84 ± 7.38	46.53 ± 7.40	**0.005**	0.19

FFM = fat-free mass; FFMI = fat-free mass index; SMM = skeletal muscle mass; BF = body fat; PhA = phase angle; TBW = total body water. Statistically significant differences (*p* < 0.05) are indicated in bold. ^1^ By means of Wilcoxon signed-rank test. ^2^ r ≥ 0.3 indicates a small effect, r ≥ 0.5 a moderate effect, and r ≥ 0.6 a large effect (12).

**Table 4:** Lin’s concordance correlation coefficients (CCC) between the InBody 970 and seca mBCA 515 devices in the total sample (*n* = 224).

	Lin’s CCC (95% CI)	Interpretation of Agreement According to [31] ^1^
FFM (kg)	0.983 (0.978–0.987)	Substantial
FFMI (kg/m^2^)	0.963 (0.954–0.972)	Substantial
SMM (kg)	0.836 (0.809–0.863)	Poor
SMM (%)	0.470 (0.413–0.528)	Poor
BF (%)	0.946 (0.934–0.959)	Moderate
PhA (°)	0.928 (0.913–0.943)	Moderate
TBW (L)	0.990 (0.987–0.992)	Almost perfect
Resistance (Ω)	0.966 (0.959–0.974)	Substantial
Reactance (Ω)	0.978 (0.973–0.984)	Substantial

FFM = fat-free mass; FFMI = fat-free mass index; SMM = skeletal muscle mass; BF = body fat; PhA = phase angle; TBW = total body water. ^1^ >0.99 almost perfect, 0.95 to 0.99 substantial, 0.90 to 0.95 moderate, <0.90 poor.

**Table 5:** Cross-tabulation of low FFMI classification based on GLIM-cited FFMI thresholds, derived from seca mBCA 515 and InBody 970 measurements in the cancer subsample (*n* = 85).

*n* (%)	Seca No Low FFMI	Seca Low FFMI	Total
**InBody** **no low FFMI**	42 (49.4)	26 (30.6)	68 (80.0)
**InBody** **low FFMI**	0 (0.0)	17 (20.0)	17 (20.0)
**Total**	42 (49.4)	43 (50.6)	85 (100.0)

FFMI = fat-free mass index. FFMI thresholds of <17 kg/m^2^ for men and <15 kg/m^2^ for women were applied, as cited in the GLIM consensus report [22,23].

**Table 6:** Cross-tabulation of altered body composition classification according to the ESPEN and EASO criteria for sarcopenic obesity, derived from seca mBCA 515 and InBody 970 measurements in the obesity subsample (*n* = 139).

*n* (%)	Seca No Altered Body Composition	Seca Altered Body Composition	Total
**InBody** **no altered body composition**	15 (10.8)	54 (38.9)	69 (49.6)
**InBody** **altered body composition**	1 (0.7)	69 (49.6)	70 (50.4)
**Total**	16 (11.5)	123 (88.5)	139 (100.0)

Altered body composition in the context of the ESPEN–EASO classification of sarcopenic obesity is defined as an increased body fat percentage together with reduced skeletal muscle mass adjusted for body weight [24]. The respective cut-offs are displayed in Table 1.

**Table 7:** Summary of subgroup effects on between-device differences (see Supplementary Table S3 for full results).

Parameter	Disease Type	Sex	Age Group
FFM (kg)	ns	↑↑ females	ns
FFMI (kg/m^2^)	↑ cancer	↑↑ females	ns
SMM (kg)	↑↑ cancer	ns	ns
SMM (%)	↑↑ cancer	↑↑ females	↑ older age groups
BF (%)	↑↑ cancer	↑↑ females	ns
PhA (°)	↑↑ obesity	↑ males	↑ older age groups
TBW (L)	↑↑ cancer	↑↑ females	ns
Resistance (Ω)	ns	↑↑ females	ns
Reactance (Ω)	ns	↑ males	ns

FFM = fat-free mass; FFMI = fat-free mass index; SMM = skeletal muscle mass; BF = body fat; PhA = phase angle; TBW = total body water. Arrows indicate the group with the larger Δ (InBody–seca). ↑ *p* < 0.05, ↑↑ *p* < 0.001, ns = not significant.

## Data Availability

The data presented in this study are available on request from the corresponding author due to institutional restrictions.

## Supplementary Materials

The following supporting information can be downloaded at: https://www.mdpi.com/article/10.3390/nu18050767/s1, Table S1: Distribution of cancer entities in the cancer subsample (*n* = 85); Table S2: Interpretation of Bland-Altman-Plots displayed in Figure 1, illustrating the agreement between the seca mBCA 515 and InBody 970; Table S3: Between-device discrepancies of the seca mBCA 515 and InBody 970 stratified by disease type, sex, and age group; Table S4: Absolute device outputs of the seca mBCA 515 and InBody 970 stratified by disease type; Table S5: Absolute device outputs of the seca mBCA 515 and InBody 970 stratified by sex; Table S6: Absolute device outputs of the seca mBCA 515 and InBody 970 stratified by age group.
