# Supplementary material for: Variability in BIA-Derived Muscle Mass Estimates: Device Choice Impacts Diagnostic Classification
Source: Nutrients. 2026 Feb 26;18(5):767. doi: 10.3390/nu18050767 (PMC12986818; doi:10.3390/nu18050767)
Supplement: Supplementary file 1 [file nutrients-18-00767-s001.zip › nutrients-4152613-supplementary.pdf]

## Supplementary Material

**Table S1:** Distribution of cancer entities in the cancer subsample (n = 85).

| Type of cancer | n (%) |
|---|---|
| Gastrointestinal cancers (gastric, colorectal, pancreatic, liver, esophageal) | 21 (24.7) |
| Lung cancer | 4 (4.7) |
| Breast cancer | 35 (41.2) |
| Other gynecological cancers (ovarian, endometrial, cervical) | 10 (11.8) |
| Urological cancers (prostate, kidney, bladder) | 1 (1.2) |
| Hematological malignancies | 6 (7.1) |
| Other solid cancers | 8 (9.4) |

**Table S2:** Interpretation of Bland-Altman-Plots displayed in **Figure 1**, illustrating the agreement between the seca mBCA 515 and InBody 970.

| Parameter | Clinical relevance of bias | Proportional bias | Heteroscedasticity | InBody vs. seca – interpretation |
|---|---|---|---|---|
| FFM (kg) | Bias = +2.13 kg. Moderate clinical relevance. | InBody shows higher values at lower FFM (kg) and lower values at higher FFM (kg) compared with seca. | Slight widening at higher means, indicating mild heteroscedasticity. | InBody yields higher values than seca. |
| FFMI (kg/m²) | Bias = +0.73 kg/m². Small to moderate clinical relevance. | InBody shows higher values at lower FFMI (kg/m²) and lower values at higher FFMI (kg/m²) compared with seca. | Even spread, indicating homoscedasticity. | InBody yields higher values than seca. |
| SMM (kg) | Bias = +5.62 kg. Large clinical relevance. | No pronounced proportional bias. | Spread increases moderately with higher means. | InBody yields consistently higher values than seca (in almost all cases) |
| SMM (%) | Bias = +6.67 %. Large clinical relevance. | InBody shows lower values at lower SMM (%) and higher values at higher SMM (%) compared with seca. | Moderate spread. | InBody yields consistently higher values than seca (in almost all cases) |
| BF (%) | Bias = -2.61 %. Small to moderate clinical relevance. | No pronounced proportional bias. | Slight heteroscedasticity in the mid-range. | seca yields higher values than InBody. |

| Parameter | Clinical relevance of bias | Proportional bias | Heteroscedasticity | InBody vs. seca – interpretation |
|---|---|---|---|---|
| PA (°) | Bias = +0.24 °. Small clinical relevance. | InBody shows lower values at lower PA (°) and higher values at higher PA (°) compared with seca. | Even spread; good consistency. | InBody yields higher values than seca. |
| TBW (L) | Bias = +1.01 L. Small clinical relevance. | InBody shows higher values at lower TBW (L) and lower values at higher TBW (L) compared with seca. | Moderate spread. | InBody yields higher values than seca. |
| Resistance (Ω) | Bias = -21.63 Ω. Small clinical relevance. | InBody shows higher values at lower Resistance (Ω) and lower values at higher Resistance (Ω) compared with seca. | Moderate spread. | seca yields higher values than InBody. |
| Reactance (Ω) | Bias = + 0.31 Ω. Clinically negligible relevance. | None. | Moderate spread. | InBody ≈ SECA |

FFM = fat-free mass; FFMI = fat-free mass index; SMM = skeletal muscle mass; BF = body fat; PhA = phase angle; TBW = total body water

**Table S3**: Between-device discrepancies of the seca mBCA 515 and InBody 970 stratified by disease type, sex, and age group.

| | Group | | | | | | | | | |
|---|---|---|---|---|---|---|---|---|---|---|
| | Disease type | | | Sex | | | Age group (years) | | | |
| Δ Inbody - seca | Cancer (n = 85) | Obesity (n = 139) | *p*-value[1] | Male (n = 78) | Female (n = 146) | *p*-value[1] | 20–39 (n = 32) | 40–59 (n = 122) | 60–80 (n = 70) | *p*-value[1] |
| FFM (kg) | 2.60 ± 1.82 | 1.85 ± 2.17 | 0.0524 | 0.79 ± 2.42 | 2.85 ± 1.42 | **<0.0001** | 2.01 ± 1.29 | 2.10 ± 2.24 | 2.23 ± 2.08 | 0.5703 |
| FFMI (kg/m²) | 0.92 ± 0.64 | 0.62 ± 0.79 | **0.0327** | 0.22 ± 0.75 | 1.00 ± 0.59 | **<0.0001** | 0.66 ± 0.43 | 0.71 ± 0.82 | 0.79 ± 0.73 | 0.3593 |
| SMM (kg) | 6.25 ± 1.01 | 5.24 ± 1.28 | **<0.0001** | 5.47 ± 1.65 | 5.70 ± 1.03 | 0.3595 | 5.52 ± 1.19 | 5.50 ± 1.31 | 5.87 ± 1.25 | 0.4391 |
| SMM (%) | 9.67 ± 2.43 | 4.84 ± 1.48 | **<0.0001** | 5.60 ± 2.74 | 7.24 ± 3.01 | **<0.0001** | 5.65 ± 2.32 | 6.33 ± 2.84 | 7.72 ± 3.31 | **0.0044** |
| BF (%) | -3.99 ± 2.90 | -1.77 ± 1.90 | **<0.0001** | -0.78 ± 2.07 | -3.59 ± 2.25 | **<0.0001** | -1.85 ± 1.17 | -2.55 ± 2.57 | -3.06 ± 2.92 | 0.0974 |
| PA (°) | 0.18 ± 0.13 | 0.28 ± 0.14 | **<0.0001** | 0.28 ± 0.15 | 0.22 ± 0.13 | **0.0005** | 0.27 ± 0.15 | 0.26 ± 0.14 | 0.19 ± 0.14 | **0.0050** |
| TBW (L) | 1.63 ± 1.04 | 0.63 ± 1.39 | **<0.0001** | 0.46 ± 1.64 | 1.30 ± 1.07 | **<0.0001** | 0.94 ± 1.02 | 0.97 ± 1.46 | 1.11 ± 1.30 | 0.8593 |
| Resistance (Ω) | -21.31 ± 16.41 | -21.82 ± 10.16 | 0.5675 | -17.01 ± 10.68 | -24.09 ± 13.27 | **<0.0001** | -24.18 ± 10.45 | -21.79 ± 13.96 | -20.17 ± 11.77 | 0.3571 |
| Reactance (Ω) | 0.26 ± 1.59 | 0.34 ± 1.46 | 0.9179 | 0.69 ± 1.14 | 0.11 ± 1.64 | **0.0038** | 0.24 ± 1.78 | 0.43 ± 1.57 | 0.15 ± 1.23 | 0.2634 |

FFM = fat-free mass; FFMI = fat-free mass index; SMM = skeletal muscle mass; BF = body fat; PhA = phase angle; TBW = total body water

Statistically significant differences ($p < 0.05$) are indicated in bold.

[1]By means of multivariable linear models.

**Table S4:** Absolute device outputs of the seca mBCA 515 and InBody 970 stratified by disease type.

| | Cancer (n = 85) | | Obesity (n = 139) | |
|---|---|---|---|---|
| | InBody | seca | InBody | seca |
| FFM (kg) | 48.41 ± 8.47 | 45.81 ± 8.99 | 64.27 ± 15.58 | 62.43 ± 16.43 |
| FFMI (kg/m²) | 16.73 ± 1.74 | 15.81 ± 1.98 | 21.78 ± 3.1 | 21.16 ± 3.51 |
| SMM (kg) | 26.24 ± 4.97 | 19.99 ± 4.97 | 35.85 ± 9.09 | 30.61 ± 9.21 |
| SMM (%) | 39.95 ± 4.62 | 30.28 ± 4.77 | 31.73 ± 4.21 | 26.89 ± 4.08 |
| BF (%) | 26.14 ± 8.22 | 30.13 ± 9.47 | 43.02 ± 7.1 | 44.79 ± 7.27 |
| PA (°) | 4.54 ± 0.62 | 4.36 ± 0.58 | 5.42 ± 0.61 | 5.14 ± 0.56 |
| TBW (L) | 35.59 ± 6.25 | 33.96 ± 6.5 | 47.32 ± 11.56 | 46.69 ± 11.94 |
| Resistance (Ω) | 609.83 ± 71.57 | 631.13 ± 76.84 | 487.78 ± 74.43 | 509.6 ± 77.57 |
| Reactance (Ω) | 48.26 ± 7.71 | 48 ± 7.8 | 45.98 ± 7.06 | 45.63 ± 7.03 |

FFM = fat-free mass; FFMI = fat-free mass index; SMM = skeletal muscle mass; BF = body fat; PhA = phase angle; TBW = total body water

**Table S5:** Absolute device outputs of the seca mBCA 515 and InBody 970 stratified by sex.

| | Male (n = 78) | | Female (n = 146) | |
|---|---|---|---|---|
| | InBody | seca | InBody | seca |
| FFM (kg) | 72.88 ± 15.31 | 72.09 ± 15.66 | 50.44 ± 7.94 | 47.59 ± 7.97 |
| FFMI (kg/m²) | 22.37 ± 3.89 | 22.15 ± 4.14 | 18.52 ± 2.63 | 17.52 ± 2.78 |
| SMM (kg) | 40.77 ± 9.03 | 35.31 ± 9.55 | 27.62 ± 4.75 | 21.92 ± 4.98 |
| SMM (%) | 38.06 ± 5.93 | 32.46 ± 4.02 | 33.13 ± 5.17 | 25.89 ± 3.08 |
| BF (%) | 31.67 ± 11.43 | 32.44 ± 11.16 | 39.26 ± 10.06 | 42.85 ± 8.74 |
| PA (°) | 5.41 ± 0.86 | 5.12 ± 0.79 | 4.91 ± 0.61 | 4.7 ± 0.56 |
| TBW (L) | 53.68 ± 11.37 | 53.23 ± 11.85 | 37.09 ± 5.88 | 35.78 ± 6.14 |
| Resistance (Ω) | 466.28 ± 81.6 | 483.29 ± 81 | 570.32 ± 79.54 | 594.41 ± 81.89 |
| Reactance (Ω) | 43.45 ± 7.13 | 42.76 ± 6.94 | 48.65 ± 6.88 | 48.54 ± 6.85 |

FFM = fat-free mass; FFMI = fat-free mass index; SMM = skeletal muscle mass; BF = body fat; PhA = phase angle; TBW = total body water

**Table S6:** Absolute device outputs of the seca mBCA 515 and InBody 970 stratified by age group.

| | 20–39 years (n = 32) | | 40–59 years (n = 122) | | 60–80 years (n = 70) | |
|---|---|---|---|---|---|---|
| | InBody | seca | InBody | seca | InBody | seca |
| FFM (kg) | 65.61 ± 20.5 | 63.59 ± 19.97 | 58.7 ± 15.27 | 56.59 ± 16.43 | 54.12 ± 11.16 | 51.89 ± 12.33 |
| FFMI (kg/m²) | 21.2 ± 4.55 | 20.54 ± 4.45 | 20.15 ± 3.65 | 19.44 ± 4.1 | 18.74 ± 2.72 | 17.95 ± 3.21 |
| SMM (kg) | 36.79 ± 12.19 | 31.27 ± 11.67 | 32.5 ± 8.89 | 27 ± 9.44 | 29.58 ± 6.6 | 23.71 ± 7.05 |
| SMM (%) | 34.45 ± 5.39 | 28.8 ± 4.16 | 34.23 ± 5.73 | 27.9 ± 4.56 | 36.1 ± 6.34 | 28.38 ± 5.03 |
| BF (%) | 38.12 ± 9.95 | 39.97 ± 9.49 | 37.91 ± 10.69 | 40.46 ± 10.31 | 33.68 ± 11.97 | 36.73 ± 11.97 |
| PA (°) | 5.57 ± 0.67 | 5.3 ± 0.63 | 5.19 ± 0.71 | 4.93 ± 0.65 | 4.67 ± 0.64 | 4.48 ± 0.58 |
| TBW (L) | 48.15 ± 15.12 | 47.21 ± 14.84 | 43.21 ± 11.36 | 42.24 ± 12.14 | 39.86 ± 8.27 | 38.75 ± 8.93 |
| Resistance (Ω) | 536.96 ± 110.57 | 561.14 ± 115.05 | 528.97 ± 96.41 | 550.77 ± 100.41 | 541.7 ± 82.46 | 561.87 ± 82.4 |
| Reactance (Ω) | 51.33 ± 6.52 | 51.09 ± 7.12 | 47.42 ± 7.44 | 47 ± 7.37 | 43.78 ± 6.36 | 43.63 ± 6.36 |

FFM = fat-free mass; FFMI = fat-free mass index; SMM = skeletal muscle mass; BF = body fat; PhA = phase angle; TBW = total body water
